# Cancer Mortality in Chinese Chrysotile Asbestos Miners: Exposure-Response Relationships

**DOI:** 10.1371/journal.pone.0071899

**Published:** 2013-08-21

**Authors:** Xiaorong Wang, Eiji Yano, Sihao Lin, Ignatius T. S. Yu, Yajia Lan, Lap Ah Tse, Hong Qiu, David C. Christiani

**Affiliations:** 1 Division of Occupational and Environmental Health, Jockey Club School of Public Health and Primary Care, the Chinese University of Hong Kong, Hong Kong, China; 2 School of Public Health, Teikyo University School of Medicine, Tokyo, Japan; 3 Huaxi School of Public Health, Sichuan University, Chengdu, China; 4 Department of Environmental Health, Harvard School of Public Health, Boston, Massachusetts, United States of America; University of Campinas, Brazil

## Abstract

**Objective:**

This study was conducted to assess the relationship of mortality from lung cancer and other selected causes to asbestos exposure levels.

**Methods:**

A cohort of 1539 male workers from a chrysotile mine in China was followed for 26 years. Data on vital status, occupation and smoking were collected from the mine records and individual contacts. Causes and dates of death were further verified from the local death registry. Individual cumulative fibre exposures (f-yr/ml) were estimated based on converted dust measurements and working years at specific workshops. Standardized mortality ratios (SMRs) for lung cancer, gastrointestinal (GI) cancer, all cancers and nonmalignant respiratory diseases (NMRD) stratified by employment years, estimated cumulative fibre exposures, and smoking, were calculated. Poisson models were fitted to determine exposure-response relationships between estimated fibre exposures and cause-specific mortality, adjusting for age and smoking.

**Results:**

SMRs for lung cancer increased with employment years at entry to the study, by 3.5-fold in ≥10 years and 5.3-fold in ≥20 years compared with <10 years. A similar trend was seen for NMRD. Smokers had greater mortality from all causes than nonsmokers, but the latter also had slightly increased SMR for lung cancer. No excess lung cancer mortality was observed in cumulative exposures of <20 f-yrs/ml. However, significantly increased mortality was observed in smokers at the levels of ≥20 f-yrs/ml and above, and in nonsmokers at ≥100 f-yrs/ml and above. A similarly clear gradient was also displayed for NMRD. The exposure-response relationships with lung cancer and NMRD persisted in multivariate analysis. Moreover, a clear gradient was shown in GI cancer mortality when age and smoking were adjusted for.

**Conclusion:**

There were clear exposure-response relationships in this cohort, which imply a causal link between chrysotile asbestos exposure and lung cancer and nonmalignant respiratory diseases, and possibly to gastrointestinal cancer, at least for smokers.

## Introduction

China, as the largest asbestos consumer and the second producer in the world, continues to mine and use chrysotile asbestos. With rapid industrialization and urbanization, the demand for asbestos has increased over time, with total production increasing from 310,000 tons in 2001 to 450,000 tons in 2009 [1]. The estimated number of occupationally exposed workers in the asbestos industry currently, excluding end users, is more than 100,000.

The relationship between exposure to chrysotile asbestos and malignant disease, such as lung cancer and gastrointestinal cancers, has been debated over the last several decades [2]–[7]. Increasing evidence supports the association with lung cancer [8]–[10] and with digestive tract cancers [11], [12]. Most early evidence for the carcinogenicity of asbestos in exposed workers was gathered in USA and Europe [13]–[16]. Some studies have provided quantitative exposure-response data for risk assessment of lung cancer in asbestos workers [17], [18]. In China, no study has been reported that addresses cancer risks in workers exposed to asbestos until the 1990s. Several studies since then have focused on asbestos textile workers and provided strong evidence for cancer risks [19]–[21]. A recent study made a quantitative estimate of individual fibre exposures and observed a clear exposure-response relationship in asbestos textile workers [22]. However, fewer studies were conducted to investigate cancer risks in exclusive chrysotile asbestos miners in China, where a huge amount of chrysotile asbestos has been mined and produced. We followed a cohort of asbestos workers in the largest chrysotile mine located in Qinghai Province, China, and recently reported a preliminary result of excessive mortality from lung cancer and respiratory diseases [23], in which no concrete exposure data were analyzed and reported. In the present report, we utilized available quantitative exposure data to make a further estimate of the risks for lung and gastrointestinal (GI) cancers, and non-malignant respiratory diseases (NMRD), in an attempt to determine exposure-response relationships in this cohort.

## Subjects and Methods

### Study Cohort

The cohort consisted of 1539 male workers employed in the chrysotile asbestos mine. The mine is China’s largest, providing one third of the total national output of chrysotile asbestos. Workers who were on the mine registry list on 1 January 1981, and employed for a minimum of one year, were recruited and followed through to 31 December 2006, irrespective of retirement. In fact, nearly half of the workers retired and left the mine during the follow-up period. Nevertheless, all of the workers had maintained a contact with the mine’s personnel department in the first two decades, as they received their pensions from the mine, as long as they were alive. Thus, the mine had kept an explicit record of workers’ vital status. The situation was changed since the last decade when workers’ pension started to be distributed from a social security system. Yet, we made a great effort to trace down each of the workers in the cohort, and got no one lost. The follow-up of the cohort generated 34,736 person-years of observation.

### Ethical Approval

The study was approved by the Human Subject Committee of the Chinese University of Hong Kong. Written consent was not obtained in the study, largely because most of the information and data were obtained from industrial or hospital records. Oral consent was obtained when we gathered smoking information and verification of occupational history from individual workers or their family members (for those deceased). We explained the purpose of the data collection to the study participants and ensured that all data were kept confidential during handling and use. The oral consents were witnessed by the administrative staff and doctors of the mine. The form of consent was approved by the Human Subject Committee.

### Data Collection

We collected data on each worker’s job type, when they first started working in the mine; number of years working at different workshops/departments, retirement date, diagnosis of asbestosis, and vital status year by year from the personnel department of the mine, where all of the information was well recorded in the first two decades. We traced down those who retried and left from the mine. There were 87 workers who were diagnosed with asbestosis by the Pneumoconiosis Diagnosis Panel using the Chinese Radiographic Diagnosis Criteria of Pneumoconiosis. We obtained information on the causes and dates of deaths from the mine hospital and other hospitals, verified with the death registry. Basically, there are consistent diagnostic criteria for cancers in China, largely based on clinical manifestations and pathological confirmation or biopsy. Smoking habits, as either smoking/ever-smoking or non-smoking, were ascertained by asking the workers (for those alive) and their spouses or next-of-kin (for those deceased) through personal contact.

### Asbestos Dust Measurements

Periodic data of total dust concentrations of different workshops were available from 1984 to 1995. The measurements were conducted based on the national criteria for dust measurement methods in the workplace (GB5748-85). Specifically, fixed point sampling in the workers’ breathing zone was applied with a flow rate of 5 litres/min (±0.1) for 4 hours. The measurements indicated that average dust concentrations in the mine changed from 800 mg/m^3^ in the 1980s to about 140 mg/m^3^ in the 1990s, with 400 to 70 times the previously applied national standard (2 mg/m^3^) [24]. In 2006, an additional 28 samples from the workers’ breathing zone in eight workshops were collected, showing particularly high dust concentrations in milling plants, ranging from 12 to 197 mg/m^3^ and with an average of 91.3 mg/m^3^ (±67.1) [23]. Based on a conservative estimate made by converting dust mg/m^3^ to fibre f/ml [25], [26], geometric mean fibre concentration was 16 f/ml in the main workshops (Table 1), which far exceeded the currently applied national occupational exposure limit of 0.8 f/ml. Average fibre concentration in other areas was up to 1.6 f/ml, also higher than the national exposure limit.

**Table 1:** Measurements of Asbestos Dust Concentrations (mg/m^3^) and Estimated Fiber Concentrations (f/ml) by Workshop in 2006.

Workshop	No. of Samples	Geometric mean (range)	
		Dust (mg/m^3^)	Fiber (f/ml)
**Main workshops**	13	47.1	15.9
Mining area	5	5.3 (0.6–17.3)	1.9 (0.5–5.6)
Milling plant 1	4	48.2 (12.5–196.7)	15.2 (4.1–63.8)
Milling plant 2	4	84.3 (31.5–128.0)	26.0 (9.9–47.4)
**Other areas**	15	3.6	1.6
Milling plant office	3	9.7 (6.3–21.3)	3.7 (2.3–6.8)
Package site	3	8.4 (4.3–13.7)	2.9 (1.6–4.5)
Maintenance area	6	2.4 (0.3–5.8)	1.1 (0.4–2.1)
Transportation site	3	2.4 (0.8–5.1)	1.1 (0.6–1.9)

Asbestos samples collected from the mine in 2006 were also analyzed to determine fibre types in a Japanese lab using the method of “JIS A 1481∶2008 determination of asbestos in building material products” with 0.1% as the limit of detection. The analysis showed no detection of tremolite, suggesting that an amphibole contamination, if any, was less than 0.1% in the asbestos samples [23].

### Cumulative Fibre Exposure Estimate

We made an estimation of cumulative fibre exposure for individual workers, using all periodically measured suspended dust concentrations at different workshops, which were available to use since 1984, and converted to fibre concentrations, using previously described methods [22], . To do this, 35 paired samples measured in 1991, with simultaneous gravimetric and membrane filter methods in main workshops in chrysotile mine, were used to develop a linear regression equation for the relationship between dust and fibre concentrations: “Dust concentration (mg/m^3^) = 3.2935×fiber concentration (f/ml) - 1.0945”, with a correlation coefficient of 0.88 (p<0.001) [26]. Next, we used the equation to calculate average airborne fibre concentrations by workshop and job title for the period from 1984 to 2006. Furthermore we estimated individual cumulative fibre exposures, expressed in fibre-years/ml (f-y/ml), as the product of estimated fibre concentration for a specific workshop and job title, and the duration of employment in each job title. There were about 400 workers who were not directly engaged in mining and milling [23], but worked in service, administrative and other jobs at the mine. Measurements of total asbestos dust concentrations were available for such environments as outside administrative buildings and service areas, ranging from 0.9 to 2.4 mg/m^3^. Even at a primary school and a hospital located at the same area, the dust concentrations in the ambient air ranged from 0.7 to 3.6 mg/m^3^. This implied that these seemingly “non-exposed workers” actually were exposed, though at a comparatively lower level. Thus, we also made an estimation of cumulative exposures for these workers. The calculation and assignment of cumulative exposures were carried out blind to workers’ vital status.

### Data Analysis

We calculated standardized mortality ratios (SMR) for lung cancer (including trachea, bronchus, lung cancer and other thoracic neoplasm, corresponding to the ICD-10 of C33, C34, C37 and C38), GI cancer including stomach cancer (C16), esophageal cancer (C15), liver cancer (C22), colorectum cancer (C18, C19 and C20), gallbladder cancer (C23, C24), and pancreas cancer (C25), and NMRD comprising asbestosis (J66), low respiratory tract diseases, emphysema and asthma (J43, Z82), pneumonia (J18), chronic bronchitis, emphysema, COPD and other respiratory diseases (J40–44), and pulmonary tuberculosis (A19). We calculated SMR for the category of the diseases, instead of more specific individual diseases, in order to enhance study power because there were a small number of deaths from each of the specific diseases. We used the 2^nd^ and 3^rd^ national survey data of cause-specific death during 1990–1992 and 2004–2005 as reference rates [29], [30]. The expected number of deaths was calculated by using 5-year age- and cause-specific mortality rates of Chinese males in 1990 and 2004, which corresponded to the time periods of 1981–1995 and 1996–2006. We did not calculate a SMR for asbestosis because of the unavailability of national mortality data for asbestosis in the general population. To determine a possible exposure-response relationship, we first stratified SMRs by employment years at entry (referring to the years a worker had been working in the mine when he entered into the cohort) and total employment years, and then stratified by estimated cumulative fibre exposures. We first categorized the cumulative fibre exposures into quartiles. However, most of the deaths from cancers centered on the high exposures, based on which we used cut points of approximately 50%, 75% and 85% to categorize the cumulative exposures, namely, <20, ≥20, ≥100 and ≥450 f-y/ml. SMRs by estimated individual cumulative exposure were stratified by smokers and nonsmokers. Furthermore, we applied Poisson regression analysis to estimate relative risks (RR) for cause-specific mortality in relation to the cumulative exposures, in which age at entry and smoking status were adjusted for. To consider the effect of latency on the outcomes of interest, we estimated cumulative fibre exposures for all selected causes with a lag of 10 years. A level of α <0.05 at two-sides was considered as statistically significance. Test of linear trend in SMRs or RRs was done by fitting models with ordinal values of each exposure category as a continuous variable. The data analysis was performed with the Statistical Package for the Social Sciences Software version 18.0 for Windows.

## Results

Table 2 represents basic demographic and vital status data of the cohort. On average, age at entry was 36 years old; workers started to work in the mine at 22 years old and their total employment was 14 years at entry of the cohort. Those smoked or ever-smoked accounted for 85%. The geometric mean of estimated individual cumulative exposures was 57.7 f-y/ml; and the arithmetic mean was 212.4 f-y/ml (without 10-year lag). In total 428 deaths (28%) were identified, of which 56 (13%) died of lung cancer, 53 (12%) of GI cancer and 84 (20%) of NMRD. There were 29 asbestosis cases in the deaths from NMRD, and one in the deaths from lung cancer.

**Table 2:** Demographic Data and Vital Status of Asbestos Miner Cohort (n = 1539), China, 1981–2006.

	Mean (SD)	N (%)
Age at entry (yrs)	36.1 (11.5)	
Age at start working (yrs)	21.7 (6.1)	
Age at end of follow up (yrs)	59.2 (10.8)	
Age at death (yrs)	61.1 (12.9)	
Years of employment at entry	14.4 (9.6)	
Total years of employment	27.3 (6.2)	
Geometric mean of fibre exposure, f-y/ml	57.7 (4.8)	
Ever-smoking (n, %)		1302 (84.6)
Asbestosis (n, %)		87 (5.7)
**Causes of death**		
All cause		428 (27.8)
All cancers*		120 (28.0)
Lung cancer		56 (13.1)
GI cancer†		53 (12.4)
NMRD‡		84 (19.6)

* Deaths of specific causes were calculated among the all deaths.

† Gastrointestinal cancers, including all cancers at digestive system.

‡ Non-malignant respiratory diseases.

Table 3 shows SMRs by employment years and age. Mortality for all of the selected causes tended to increase with employment years at entry, with the clearest trend seen in lung cancer, in which those with 20 or more employment years had a 7.5-fold increase in mortality over that expected. A similar trend, though with lower magnitude, was observed in NMRD, all cancers and all causes. When stratified by the total employment years, mortality for either lung cancer or NMRD with 20 or more years was more than double that in less than 20 years, while over 30 years no further increases in mortality were observed. Mortality, especially from lung cancer and NMRD, increased with age at entry.

**Table 3:** Standardized Mortality Ratios (SMRs) by Employment Years and Age at Entry in Chrysotile Miner Cohort (n = 1539), China, 1981–2006.

	All causes		All cancers		Lung Cancer		GI cancer		NMRD‡	
	O*	SMR (95%CI)†	O	SMR (95% CI)	O	SMR (95% CI)	O	SMR (95% CI)	O	SMR (95% CI)
Employment years at entry										
<10	122	1.81(1.51,2.16)	19	0.98(0.63,1.53)	6	1.40 (0.64, 3.06)	10	0.61(0.33,1.12)	15	1.21(0.74,2.01)
≥10–<20	97	2.56(2.10,3.13)	36	2.98(2.15,4.12)	13	4.92 (2.88, 8.43)	19	1.90(1.22,2.97)	13	2.19(1.28,3.74)
≥20	209	3.04(2.66,3.48)	65	3.02(2.37,3.85)	37	7.46(5.41,10.28)	24	1.74(1.17,2.59)	56	5.66(4.36,7.35)
Total employment years										
<20	171	2.15(1.85,2.50)	37	1.62(1.17,2.23)	17	3.26(2.04, 5.23)	16	1.00(0.62,1.62)	25	1.98(1.34,2.93)
≥20–<30	170	2.70(2.32,3.14)	58	2.94(2.27,3.80)	31	7.05(4.96,10.0)	25	1.65(1.12,2.43)	42	4.08(3.02,5.51)
≥30	87	2.74(2.22,3.38)	25	2.39(1.62,3.54)	8	3.54 (1.79, 6.99)	12	1.33(0.76,2.33)	17	3.22(2.01,5.16)
Age at entry§, yrs										
<30	42	2.32(1.72,3.14)	3	1.37(0.48,4.05)	0	0/0.28	3	0.68(0.23,1.99)	2	0.46(0.23,1.99)
≥30–<40	81	1.85(1.49,2.30)	24	1.43(0.96,2.13)	7	1.18 (0.57, 2.43)	13	0.84(0.49,1.44)	10	1.19(0.65,2.20)
≥40	305	2.72(2.43,3.04)	93	2.73(2.23,3.35)	49	7.27 (5.50, 9.61)	37	1.83(1.32,2.52)	72	5.16(4.10,6.50)

* observed number of deaths.

† 95% confidence interval.

‡ non-malignant respiratory disease.

§ age of workers when they entered into the cohort on January 1, 1981.

Overall SMRs from all of the selected causes were significantly higher, particularly from lung cancer and NMRD, with nearly 5- and 3-fold SMRs, respectively, that expected (Table 4). When SMRs were stratified by smoking status, slightly excess mortality, though insignificantly, was seen for lung cancer (SMR 1.46; 95% CI, 0.50, 4.30) in nonsmokers. In smokers, there was significantly increased mortality for either lung cancer (SMR 5.40; 4.13, 7.07) or NMRD (SMR 3.33; 2.67, 4.15), which was considerably greater than in nonsmokers. No increased mortality for GI cancer was observed in nonsmokers, but a slight and significant increase was seen in smokers (SMR 1.38; 1.04, 1.84).

**Table 4:** SMRs in All and by Smoking Status in Chrysotile Miner Cohort in China during 1981–2006, China, 1981–2006.

	Total (n = 1539)			Nonsmokers (n = 237)			Smokers (n = 1302)		
	O*	SMR	95% CI†	O	SMR	95% CI†	O	SMR	95% CI
All causes	428	2.46	2.23, 2.70	36	1.20	0.89, 1.71	392	2.71	2.45, 2.99
All cancers	120	2.26	1.89, 2.71	12	1.23	0.77, 2.35	108	2.47	2.03, 2.96
Lung cancer	56	4.69	3.61, 6.09	3	1.46	0.50, 4.30	53	5.40	4.13, 7.07
GI cancer	53	1.32	1.01, 1.73	7	1.01	0.49, 2.09	46	1.38	1.04, 1.84
NMRD‡	84	2.98	2.41, 3.69	6	1.27	0.58, 2.77	78	3.33	2.67, 4.15

* observed number of deaths.

† 95% confidential interval.

‡ non-malignant respiratory disease.

When SMRs were stratified by estimated cumulative fibre exposures in smokers (Table 5), mortality from all selected causes tended to increase with exposure levels, with the clearest gradient in lung cancer. While no increased mortality was observed in the category of <20 f-y/ml, there was significant excess mortality from lung cancer at the higher levels, with SMRs ranging from 4.4 (95% CI, 2.5, 7.7) at ≥20 f-y/ml, to 10.9 (6.7, 17.7) at ≥100 f-y/ml and 18.7 (12.1, 28.9) at ≥450 f-y/ml. For mortality from NMRD, there was a similarly clear exposure-response relationship, with a nearly 10- fold SMR at the highest exposure level, compared with the lowest level. An exposure-response trend was also seen for all cancers and all causes. No excess mortality for GI cancer was found at the level of <100 f-y/ml, but a significant increase was observed at ≥100 f-y/ml. When a similar analysis was carried out in nonsmokers, in whom the total number of subjects and the number of deaths in each category were small, an exposure-response trend was less clear shown in each of outcomes (Table 6). However, significantly increased mortality from lung cancer was seen at the level of ≥100 f-y/ml and above, in spite of wide conference intervals. In addition, excessive mortality from GI cancer and NMRD was also displayed at the level of ≥100 f-y/ml and above.

**Table 5:** Standardized Mortality Ratios (SMRs) by Cumulative Fibre Exposures (f-y/ml)* in Smokers in Chrysotile Miner Cohort (n = 1302), China, 1981–2006.

	All causes		All cancers		Lung Cancer		GI cancer		NMRD§	
	O†	SMR (95%CI)‡	O	SMR (95% CI)	O	SMR (95% CI)	O	SMR (95% CI)	O	SMR (95% CI)
<20	109	1.58(1.31,1.11)	15	0.73 (0.44,1.21)	5	1.10 (0.47, 2.58)	7	0.42(0.20, 0.87)	18	1.53(0.97, 2.42)
≥20	69	1.73(1.36,2.18)	20	1.64(1.06,2.53)	12	4.41 (2.52, 7.71)	7	0.78(0.38, 1.61)	12	1.98 (1.13, 3.46)
≥100	89	4.30(3.50,5.29)	30	4.75(3.33, 6.78)	16	10.88(6.70,17.68)	13	3.41(1.99,5.84)	23	7.52(5.01,11.28)
≥450	126	8.20(6.89,9.76)	44	8.73(6.50,11.72)	20	18.69(12.10, 28.87)	19	5.04(3.23,7.87)	25	9.77(6.61,14.42)
P for trend	<0.001		<0.001		<0.001		<0.001		<0.001	

* a lag of 10 years was considered in calculating cumulative fibre exposure.

† observed number of deaths.

‡ 95% confidence interval.

§ non-malignant respiratory diseases.

**Table 6:** Standardized Mortality Ratios (SMRs) by Cumulative Fibre Exposures (f-y/ml)* in Nonsmokers in Chrysotile Miner Cohort (n = 237), China, 1981–2006.

	All causes		All cancers		Lung Cancer		GI cancer		NMRD§	
	O†	SMR (95% CI)‡	O	SMR (95% CI)	O	SMR (95% CI)	O	SMR (95% CI)	O	SMR (95% CI)
<20	14	0.83(0.50,1.40)	5	0.95 (0.41, 7.75)	1	0.83 (0.15, 4.68)	3	0.72 (0.25,0.13)	1	0.37 (0.07, 2.09)
≥20	8	0.93(0.47,1.84)	1	0.41 (0.07, 2.30)	0	0/0.10	1	0.53 (0.09, 2.98)	2	1.39 (0.38, 5.06)
≥100	5	3.31(1.41,7.75)	2	4.55(1.25,16.58)	1	10.00(1.77,56.65)	1	5.00(0.88,28.33)	1	5.26(0.93,29.82)
≥450	8	3.49(1.77,6.89)	3	4.00(1.36,11.76)	1 6.25(1.10, 35.41)		2	3.03(0.83,11.05)	2	5.13(1.41,18.70)
P for trend	<0.001		<0.001		<0.01		<0.001		<0.001	

* a lag of 10 years was considered in calculating cumulative fibre exposure.

† observed number of deaths.

‡ 95% confidence interval.

§ non-malignant respiratory diseases.

In the Poisson models, in which smokers and non-smokers were combined, but smoking and age were adjusted for (Table 7), the exposure-response trend for GI cancer turned out to be clearer, with relative risks of 1.4 (95% CI, 0.6, 3.7) at ≥20, 5.1 (2.2, 11.9) at ≥100 and 8.1 (3.8, 17.2) at ≥450 f-y/ml. Meanwhile, clear exposure-response relationships with lung cancer and NMRD persisted. In the models, smoking was associated with a 4-fold risk for lung cancer and a 2-fold risk for nonmalignant respiratory diseases, relative to nonsmoking.

**Table 7:** Relative Risks* (95% CI)† for Cause-specific Death Risks in Relation to Cumulative Fibre Exposures (f-y/ml)‡ in Chrysotile miner Cohort (n = 1539), China, 1981–2006.

	All causes	All cancers	Lung Cancer	GI cancer	NMRD§
Exposure level					
<20	1.00	1.00	1.00	1.00	1.00
≥20	1.09 (0.82, 1.45)	1.77 (0.96, 3.27)	3.41 (1.29, 8.97)	1.44 (0.57, 3.66)	1.55 (1.38, 1.74)
≥100	2.48 (1.88, 3.28)	4.80 (2.72, 8.48)	7.40 (2.91, 18.80)	5.13 (2.20, 11.93)	4.26 (3.80, 4.77)
≥450	4.06 (3.17, 5.19)	9.28 (5.46, 15.77)	14.69 (5.75, 37.48)	8.07 (3.79, 17.19)	9.36 (8.47, 10.34)
p for trend	<0.001	<0.001	<0.001	<0.001	<0.001
Age	1.11 (1.09, 1.12)	1.14 (1.11, 1.16)	1.18 (1.15, 1.23)	1.10 (1.06, 1.13)	1.15 (1.12, 1.18)
Smoking	1.98 (1.40, 2.80)	1.56 (0.84, 2.92)	4.22 (1.02, 17.44)	1.05 (0.47, 2.35)	2.08 (0.90, 4.79)

* using Poisson regression models with adjustment for smoking and age at entry.

† 95% confidence interval.

‡ a lag of 10 years was considered in calculating cumulative fibre exposure.

§ non-malignant respiratory disease.

## Discussion

In this study, we followed an asbestos miner cohort from the largest Chinese chrysotile asbestos mine for 26 years, and observed some interesting findings: there were nearly 5- and 3-fold SMRs that expected for lung cancer and nonmalignant respiratory diseases, respectively. We further looked into employment duration and estimated cumulative fibre exposures, as indicators for exposures of the workers, and observed clear exposure-response relationships with lung cancer and NMRD.

Although the workers with more employment years at entry into the cohort were found to have higher mortality for the selected causes, the most prominent gradient with employment years at entry was seen for lung cancer. A similar gradient, but of lower magnitude, was observed in nonmalignant respiratory diseases. On the other hand, there was a less clear trend with total employment years. The results implied that the variable of employment years at entry reflected better workers’ exposures than the total exposure years. This might be explained by the fact that the dust concentrations in the mine were higher and more stable in the earlier years, when no control measures and effective personal protective equipments were available [23]. We also found that mortality from all selected causes increased with age at entry. This is not surprising because age is a well recognized risk factor for mortality from these causes. Also, those with older ages at entry tended to have longer employment years in the mine.

In this study, we examined the relationships between mortality from lung cancer and other causes and estimated cumulative fibre exposures. In China, asbestos fibre measurements have barely been conducted in either asbestos mines or factories, largely because a national standard for allowable fibre exposure did not exist until recently. We utilized available dust data and individual working years at different workshops to make estimates of cumulative fibre exposures for individual workers. Previous studies using similar approaches suggested that fibre exposures converted from dust concentrations could reasonably reflect workers’ exposure levels [27], [28]. One recent study [31] using converted cumulative fibre exposures observed an exposure-response relationship with malignant mesothelioma and lung cancer, as well as for colorectal cancer. Another recent study conducted among Chinese asbestos textile workers used the same approach and suggested significant exposure-response relationships with lung cancer and asbestosis [22]. We observed clear exposure-response relationships with mortality from lung cancer and nonmalignant respiratory diseases, especially in smoking asbestos miners. It is interesting to note that no excess mortality for lung cancer was found at the exposure level below 20 f-y/ml. However, the mortality significantly increased at a higher level, showing a clear gradient, with SMRs of 4-, 11- and 19-times at ≥20 f-y/ml, ≥100 f-y/ml and ≥450 f-y/ml, respectively. For nonmalignant respiratory diseases, only slightly excess mortality was observed at the lowest exposure level, but significantly increased mortality was found at higher levels. Although there was less clear exposure-response gradient in nonsmokers, possibly due to a small number of nonsmokers and deaths in each category, we observed significantly increased mortality from lung cancer at ≥100 f-y/ml and ≥450 f-y/ml. The results indicated the increased mortality associated with asbestos exposure.

It is noteworthy that most of the workers in the cohort were long term smokers. In China, smoking is common in the general male population, accounting for about 65%, and even more prevalent in manual workers. For example, up to 78% of workers smoked in a recently reported asbestos textile worker cohort [21]. It is not surprising that smoking might have contributed significantly to mortality for lung cancer and nonmalignant respiratory diseases, as indicated by the results that the smokers had 3.7-fold SMR for lung cancer and 2.6-fold SMR for nonmalignant diseases, compared to the nonsmokers. Smoking is likely to act as effect modifier on the asbestos-lung cancer association. Our previous analysis with the same data suggested the interaction of smoking and asbestos exposure though marginally statistically significant (possibly due to a small number of non-smokers) [23]. Nevertheless, the observed excess mortality cannot be explained by smoking alone, given the consistent exposure-response relationships with either exposure duration or estimated cumulative exposures. The clear exposure-response relationships persisted in the multivariate models where the smoking effect was taken into account. Moreover, there was significantly increased mortality in the nonsmokers at the high exposure levels, in spite of the small number of non-smokers in this cohort.

The associations of exposure to chrysotile asbestos with lung cancer and malignant mesothelioma have been a pressing topic for a long time [2], [4], [5], [7]. A positive association, especially with lung cancer, has been supported by increasing amounts of data for chrysotile [32]. For example, a study in South Carolina of asbestos textile workers exhibited two-fold SMRs for lung cancer compared with the general population, with an exposure-response trend [33]. A cohort study in North Carolina textile plants also reported nearly doubled SMR for lung cancer and an increased risk with cumulative fibre exposure [10]. Another recent prospective cohort study reported over three-fold risk for lung cancer in Chinese asbestos textile workers compared with non-exposed workers [34]. That study also found a clear exposure-response trend in either smokers or nonsmokers by using different exposure categories as a surrogate for worker exposure levels. In terms of asbestos miners, most information was provided in Quebec cohort studies, which included male workers employed for at least 1 month in a chrysotile mine and mills [17], [18], [35]–[37]. One study analyzed mortality data observed from 1976 to 1988 and showed 40% more than expected mortality from lung cancer [17]. The study also provided information on exposure-response relationships, in which available dust concentrations were used to assign each man’s dust exposure, expressed as million particles per cubic foot×years (mpcf.y). Significantly increased mortality was observed in those with an exposure of 300 mpcf-y or above, with greatest mortality (3.04; 95% CI 1.90, 4.60) in the highest exposure (≥1000 mpcf.y). Compared with the Quebec miner cohort, this Chinese miner cohort was exposed to a much higher level of asbestos overall, based on the facts that an average employment was 14 years at entry, and dust/fibre measurements at workplace were even higher than the highest exposure category in Quebec chrysotile mine [17], [38]. Not surprisingly, the excess mortality rates observed in this cohort were higher than those reported from the Quebec studies.

Another interesting finding from this cohort was increased mortality from gastrointestinal cancer. In smokers, there was a 3.4-fold mortality rate at ≥100 f-y/ml, and 5.0-fold mortality at ≥450 f-y/ml, both of which were statistically significant. Increased mortality from GI cancer at the similar exposure levels was also detected in nonsmokers, though insignificant. This was different from lung cancer in which a significantly increased mortality was found at a lower level (≥20 f-y/ml). An exposure-response trend for GI cancer was clear, especially after age and smoking were adjusted for, with an 8-fold risk in the highest exposure. There was generally limited evidence for the association of asbestos exposure with GI cancers; and existing studies reported inconsistent results in previous studies [11], [12], [39]–[41]. In the Quebec miners, a substantial excess mortality from gastrointestinal cancers was reported, though the authors suggested that some other factors, such as environments, perhaps selective, might also operate [35]. Although we could not rule out such an explanation, a heavy exposure to asbestos might also be a part of the explanation, given a clear exposure-response trend. The result showing a significantly increased mortality from gastrointestinal cancers at a higher exposure level than that observed for lung cancer was consistent with the reality that a smaller quantity of asbestos would be absorbed through ingestion than through inhalation. As previously reported, on average an individual would ingest about one twentieth of the amount of asbestos that they inhale [42].

One of the strengths of this study is the fact that the studied subjects were exposed to relatively pure chrysotile. Because of the special geological features and remote location of the mine, the workers usually stayed with the mine for a lifetime and had little opportunity to change their jobs. This made the possibility that these workers were exposed to other occupational carcinogens slim. Another advantage of this study is that smoking information is available, which was seldom the case in many previous studies. We observed higher risks of mortality from lung cancer and nonmalignant respiratory diseases in smokers than in nonsmokers. Nevertheless, we were not able to assess adequately mortality for the selected causes associated with asbestos exposure alone due to the small number of nonsmokers in the cohort, in spite of that the data indicated increased mortality from lung cancer and other causes at the high exposure levels. Therefore, the results obtained from the study may not be generalized to those asbestos workers who never smoked. In addition, we need to point out that we did not identify malignant mesothelioma cases in the cohort. It has been noted that the relatively high levels of asbestos exposure being experienced and the fact that China is the world’s greatest user of chrysotile are not reflected in the reported incidence of malignant mesothelioma [43]. The diagnosis of malignant mesothelioma is difficult, especially in China. Although asbestos-related mesothelioma has been defined as occupational disease officially since the 1980s, cases of malignant mesothelioma were seldom diagnosed and reported. Some cases might be misdiagnosed as lung cancer. Therefore, we could not rule out the possibility that the cases of lung cancer observed in the cohort contained a few cases of malignant mesothelioma. Another major limitation was the method of estimating individual fibre exposures by converting from periodically measured dust concentrations. We acknowledge that the estimation might have led to exposure misclassification. However, the misclassification was likely to be non-differential, meaning that it would apply to everyone, rather than only to workers with a certain level of exposure or workers with certain outcomes of interest. When calculating individual fibre exposures, the statistician was blind to the vital status of the subjects. A non-differential misclassification of individual exposures would lead to an attenuation of the associations under study. On the contrary, the clear exposure-response relationships detected in this study suggested that the estimated individual fibre exposures reflected the workers’ exposure levels to a large extent. The study further demonstrated strong associations of exposure to chrysotile asbestos with lung cancer and non-malignant respiratory diseases, and possibly with gastrointestinal cancer, at least for smokers.
